# Tarnished gold—the “standard” urine culture: reassessing the characteristics of a criterion standard for detecting urinary microbes

**DOI:** 10.3389/fruro.2023.1206046

**Published:** 2023-07-11

**Authors:** Linda Brubaker, Toby C. Chai, Harry Horsley, Rajvinder Khasriya, Robert B. Moreland, Alan J. Wolfe

**Affiliations:** ^1^ Department of Obstetrics, Gynecology, and Reproductive Sciences, Division of Female Pelvic Medicine and Reconstructive Surgery, University of California San Diego, La Jolla, CA, United States; ^2^ Department of Urology, Boston University School of Medicine and Boston Medical Center, Boston, MA, United States; ^3^ Department of Renal Medicine, Division of Medicine, University College London, London, United Kingdom; ^4^ Eastman Dental Institute, Department of Microbial Diseases, University College London, London, United Kingdom; ^5^ Department of Microbiology and Immunology, Stritch School of Medicine, Loyola University Chicago, Maywood, IL, United States

**Keywords:** diagnostics, microbiome, urinary tract infection, urine culture, urobiome, uropathogen

## Abstract

Diagnosis and treatment of urinary tract infections (UTIs) remains stagnant. The presumption that a patient either has a UTI or does not (binary choice) is inappropriately simplistic. Laboratory diagnostic tests have not advanced for decades. The goal of UTI treatment has not been rigorously defined and may increase the prescription of potentially harmful, inappropriate antibiotics. Despite the high incidence of UTI diagnoses, the high cost of UTI treatment, and increasing concerns associated with antimicrobial resistance, the development of novel and more accurate UTI tests has not been considered a priority, in part due to the general perception that current UTI care is already sufficient. In this review, we discuss the importance of improving UTI diagnostic testing to improve treatment outcomes. We discuss the problems associated with UTI diagnosis. Urinary microbes are alive and exist in both healthy and symptomatic individuals—urine is not sterile. We specifically outline the limitations of standard urine culture methods used by clinical microbiology laboratories, explaining clearly why such methods cannot be considered to be the “gold standard,” as standard culture methods underreport most of the urinary tract microbes, including some acknowledged and many emerging uropathogens. We do not recommend abandonment of this test, as no universally accepted substitute yet exists. However, we strongly encourage the development of new and improved diagnostic tests that can both improve outcomes and preserve antibiotic stewardship.

## Introduction

For decades, a urinary tract infection (UTI) has been considered a dichotomous diagnosis. Despite efforts to standardize the diagnostic criteria, they remain problematic (1, 2). More importantly, the goal of UTI treatment has not been rigorously defined (3, 4).

There is an increasing appreciation of the harms associated with indiscriminate use of antibiotics associated with the lack of diagnostic clarity. These harms include long-term changes to the human microbiome, especially within the gut, but also with the possibility of deleterious effects to the microbiomes of the urinary tract (urobiome) and the vagina through the eradication of beneficial microbes that would normally play a role in preventing clinically important infections and the selection of undetected antibiotic-resistant microbes (5, 6).

Despite the high incidence of UTI diagnoses, the high cost of UTI treatment, and the increasing concerns associated with microbial resistance, the development of novel and more accurate UTI tests has not been considered a priority, largely due to other compelling public health concerns (e.g., the COVID-19 pandemic), but also due to the general perception that current UTI care is “adequate” (7). In this review, we outline the importance of improving the evidence for a rigorous understanding of UTI diagnostic testing, as well as for the use of these tests in the assessment of UTI treatment outcomes. We specifically outline our rationale for raising awareness of the limitations of standard urine culture (SUC) methods typically used by clinical microbiology laboratories.

## Defining urinary tract infections in the urobiome era

There exists a significant body of animal research into the physiologic changes associated with UTIs; however, most of this work predates the discovery of the urobiome, the recently discovered microbial community of the urinary tract (8). The definition of acute uncomplicated cystitis (often referred to as “UTI”) is typically a clinical diagnosis, augmented by the flawed interpretation of various tests, including SUC and urinalysis. For UTI testing, however, the evidence required to assume the “criterion standard” is unclear. The diagnosis of a UTI is especially problematic when there is a mismatch between patient symptoms and SUC test results. This is especially problematic for patients with frequent or recurrent UTIs, because false “negative” results do not align with continued symptoms (9) and cultures do not affect outcome in primary care (10).

To date, there is negligible information about the human urobiome during UTIs and, importantly, during UTI resolution and recovery. Clinically, symptoms are the reason to treat and relief of symptoms is the goal of care. Unfortunately, the proxy of “no symptoms” is often equated with recovery. In fact, many clinicians inappropriately equate a report of “no growth” with “no infection,” because they do not appreciate the limitations of SUC. Because of these limitations, research that informs urobiome vulnerability, the spectrum of urobiome perturbations, the relationship between symptoms and urobiome community changes, the urobiome consequences of long- and short-term effects of antibiotics, and the predictors of successful restoration of a healthy human urobiome are urgently needed.

## Current clinical diagnostic algorithms

In the urobiome era, current clinical definitions of UTIs are very problematic. Furthermore, no tests adequately account for the host’s response outside of the rudimentary assessment for pyuria. Thus, increased scientific rigor is warranted to refine diagnostic testing for UTI based on advances in technology, new culture-independent techniques, the growing awareness of the complexity of the human microbiome, the low biomass of voided urine (termed the urogenital microbiome), and the host’s response to disturbances of the microbiome (8).

UTI antibiotics may be prescribed based on symptoms only, as well as “positive” testing. Although a less common problem, antibiotics also may be withheld (perhaps erroneously) for “negative” tests, despite symptoms. Clinicians rely heavily on the clinical presentation of patients, segregating patients into “uncomplicated” versus “complicated” cases to plan UTI testing and treatment. In the vast majority of patients, UTI diagnosis commonly leans toward one of two approaches. In the uncomplicated patient with “typical UTI” symptoms (often young women), empiric antibiotic treatment is common. Some clinicians incorporate minimal office-based testing, such as dipstick urinalysis, despite the significant limitations of dipstick testing (11). Underlying this approach is the desire to quickly initiate therapy to relieve patient suffering and minimize cost. Urine cultures are rarely used in this clinical scenario. The other approach incorporates urine culture testing, either via a reflex approach following an abnormal laboratory-based urinalysis or without preliminary urinalysis.

## Problems with current urinary tract infection clinical care algorithms

Improved patient care relies on the continuous incorporation of new evidence and modification of care algorithms. Currently, sufficient evidence exists to modify the UTI clinical care algorithm. Unfortunately, the “goal” of UTI treatment is not rigorously defined and may include only symptom relief and/or microbial “eradication” without formal testing.

The existing clinical diagnostic algorithms, described earlier, foster inappropriate antibiotic use. While clinicians are aware of the need to reduce antibiotic use, they wish to relieve suffering for their individual patient and often prescribe empiric therapy based on “usual microbes and local antibiograms.” Although there may be symptom relief, the clinician has no insight into disruptions to the urobiome (or other microbiomes, especially the gut): the selected antibiotic may have disrupted beneficial microbes or selected for previously minor but antibiotic-resistant uropathogens. This is particularly problematic within elderly care, where *Clostridioides difficile* infection is a major concern (12, 13).

The specific symptoms attributed to UTIs are highly dependent on clinical context. To support a UTI diagnosis, clinicians anticipate abrupt changes in comfort and normal functions of the lower urinary tract. In patients without symptoms, a diagnosis of “asymptomatic bacteriuria,” based on the SUC of voided urine, is problematic. “Asymptomatic bacteriuria” may not be the best term since bacteria in healthy urine is the rule, not the exception. This misperception leads to excess testing and antibiotic treatment (7, 14). Proponents of the “no antibiotic until positive urine culture” approach suggest that this strategy is more rigorous and aligned with improved antibiotic stewardship. However, even preliminary SUC results can take several days. Patient suffering is often prolonged while the clinician (who is likely trying to do the right thing) waits several days for a SUC result (and/or the laboratory’s assessment of microbial antibiotic sensitivity) in order to select a treatment antibiotic. Urinary analgesics, such a phenazopyridine (available over the counter or by prescription) and Uribel (a prescription medication of five drugs: hyoscyamine, methenamine/phenyl salicylate, sodium phosphate, and methylene blue), may be prescribed to relieve bladder pain or discomfort. However, specific anti-inflammatory medications are not routinely recommended. Other than with bladder analgesics, symptoms are not typically treated, despite evidence that anti-inflammatory medications may be clinically helpful (15). The time to SUC result availability is too long, whereas quicker “proxy” testing (e.g., standard urine dipstick) is limited and may delay appropriate treatment selection.

While clinicians currently trust SUC results to guide therapy, the laboratory often will report only the sensitivity profile of the predominant or only detected microbe. This microbe may play a role in producing the symptoms or in urobiome alterations; however, clinicians are not given information about the other microbes that are present and that may be impacting the patient’s clinical status. Conceivably, a clinician’s treatment plan could change if the report indicated the presence of three uropathogens, instead of just the most predominant (e.g., *Escherichia coli*).

Furthermore, the current diagnostic and treatment focus has been on the “pathogenic” microbes, which are considered in a dichotomous manner (i.e., uropathogen versus not). Pathogens can be divided into two categories: professional and opportunistic. Examples of professional pathogens are *Vibrio cholerae, Yersinia pestis*, and SARS CoV-2. Very few pathogens of the urinary tract are professional pathogens. Instead, most—including uropathogenic *E. coli*—are “opportunistic” pathogens, producing symptoms in some circumstances but not in others (16, 17). Those circumstances can include the immunological status of the patient and the composition of the urobiome itself. Thus, clinicians should have a broad understanding of the health of the entire urobiome community, including the presence of beneficial microbes as well as “opportunistic” pathogens. It is a disservice to provide clinicians with only a (delayed)! snapshot of one common uropathogen, which is the typical report from the SUC.

Instead, modern UTI treatment plans should be based on rigorously developed rapid testing. Point-of-care, or at least rapid, testing methods that account for the entirety (or majority) of the urobiome are long overdue for UTI diagnosis and treatment planning. Such testing could facilitate symptom relief with a sound antibiotic recommendation, if appropriate, along with bladder analgesics and anti-inflammatory medications. Note, however, that overreliance on current commercially available DNA sequencing tests is problematic, as it is unfamiliar to most clinicians. The listing of many microbes, which is common in urobiome sequence-based reports, may stimulate overtreatment to eradicate microbes, merely based on presence without an overall strategy to restore urobiome health. Treatment algorithms based on these reports simply do not yet exist.

Modern UTI treatment plans should also incorporate the host’s response. Currently, patient-reported symptoms represent the primary host response used in the evaluation and management of UTIs. An objective measure of host response to a UTI is pyuria (i.e., the simple quantification of leukocytes by urinalysis). Although UTI research has shifted toward the immunological response to certain uropathogens, especially uropathogenic *Escherichia coli*, those studies have almost exclusively relied upon mouse models, which may not fully represent the human immune response (18). Furthermore, no research has been performed on immunological responses to the healthy urobiome. Future UTI testing should integrate microbiological and objective biological host-response data to ensure better patient outcomes, while maintaining antibiotic stewardship.

## How we got here: a short history

Historically, the urinary tract has been studied with the belief that the bladder was devoid of bacteria. The longstanding human medicine dogma that “urine is sterile” dates to the mid-1800s, an era when all bacteria were considered pathogens and microbiology was in its infancy. Louis Pasteur’s attempt to disprove spontaneous generation of microbial life included the demonstration that a flask of boiled urine exposed to air turned cloudy but a flask of boiled urine in a sealed container did not (19, 20). Two decades later, Roberts concluded that “fresh and healthy urine is perfectly free from bacteria or other minute organisms” (21). Limited by the tools available at the time, he misinterpreted his data; current evidence shows that many urinary microbes do not grow under ambient atmospheric conditions (150 mm Hg PO_2_), which is one reason why SUC fails to detect many urinary microbes (22).

Throughout the history of medicine, there have been discoveries and reports that have challenged the “urine is sterile” dogma with accumulating evidence to the contrary (23–26). These insights were accelerated by the research teams who pioneered the human microbiome project over the past two decades (60). As such, there is a growing appreciation that the symbiotic co-existence of beneficial microbes and humans may be necessary for health, with disease resulting, in part, from a disruption of the normal eubiotic state (27).

The first documented use of a urine culture colony count to assess human urinary disease was in the 1950s, when Edward Kass proposed a threshold [10^5^ colony-forming units per milliliter (CFU/ml)] to detect patients with an upper UTI (pyelonephritis) (28). Based on his analysis of urine samples obtained from both symptomatic and asymptomatic women with diabetes or a cystocele or who were pregnant, he proposed that this threshold distinguished contamination from pyelonephritis (29). His method is the basis of the current SUC test, which was inappropriately generalized without rigorous evidence as a clinical test for UTI diagnosis (cystitis). However, multiple subsequent studies have provided evidence that the ≥10^5^ CFU/mL threshold alone is insufficient to diagnose clinically relevant cystitis (30–33).

An opportunity to debunk the “urine is sterile” dogma occurred when Rosalind Maskell observed slow-growing microbes (species of the genera *Corynebacterium, Lactobacillus*, and *Streptococcus*) in urine obtained from patients with UTI-like symptoms, but negative SUCs (34). These and other slow-growing microbes require growth conditions that differ from those of SUC. Maskell concluded that SUC was insufficient for diagnosis of many urinary disorders. Unfortunately, her conclusion was repudiated and ignored, although current evidence supports her conclusion.

The preponderance of evidence generated to date has focused on bacteria, although there is evidence that archaea, fungi, and viruses (both human and bacterial) are present in the human urinary tract, including the bladder. Because the literature concerning non-bacterial microbes of the urinary tract is extremely sparse, this review will focus primarily on the evolving evidence for the bacterial urobiome.

## Discovery of the urobiome

In 2010, Nelson and co-workers reported a study comparing the bacteria detected in voided urine obtained from young males with and from those without a sexually transmitted infection (STI). Using 16S rRNA gene sequencing, they identified multiple bacterial genera. All urine samples regardless of the donor’s STI status contained bacteria; however, the ones detected in males with an STI were predominantly fastidious, anaerobic, and/or uncultivated, and these were generally not present in males without an STI. While the authors focused on the difference between males with and without an STI, a major finding was that voided urine from young males contained bacteria (35). A second paper, published a year later by the same team, used 16S rRNA gene sequencing to compare the microbiomes of paired voided urine and urethral swabs provided by young males. The paired urines and swabs resembled each other, and thus the authors concluded that voided urine could be used to characterize the urethral microbiome of young males (36). A year later, Wolfe and colleagues extended these results to adult women, applying 16S rRNA gene sequencing to samples obtained from adult females undergoing urogynecological surgery. On the day of surgery, they obtained urine by three means: midstream void, suprapubic aspiration, and transurethral catheterization. At the same time, they also obtained a vaginal swab. As a control for suprapubic aspiration, the investigators also collected a suprapubic skin swab and a suprapubic needle stick (sham, without bladder entry). Some of the voided urine sample was sent to the clinical microbiology laboratory. In all but one case, the SUC result was “no growth,” and considered negative. The microbiomes of the catheterized and aspirated samples resembled each other and often did not resemble the voided sample, which often resembled the vaginal swab. The authors came to two major conclusions: (1) since the aspirated sample did not look like the skin swab or sham needle stick and it bypassed the vulva, it should be considered to represent the microbiome of bladder urine, and (2) since the microbiome of the catheterized sample resembled the aspirated sample, transurethral catheterization could be used to sample the bladder urine microbiome (37). In the same year, another team used 16S rRNA gene sequencing to assess the microbiomes of voided and catheterized urines of males and females with and without neurogenic bladder. They concluded that “healthy” urine is not sterile (38). A few other early studies came to the same conclusion (39–41). More recently, several reports have added confidence to this conclusion. For example, Vaughan used 16S rRNA gene sequencing to evaluate catheterized urines from age-matched women with and without recurrent UTI. Using a Bayesian analysis, the authors found that these cohorts differed primarily in the presence of strict anaerobes, which SUC cannot detect (42). This result was reinforced by Perovic and colleagues, who used 16S rRNA gene sequencing to detect bacteria in midstream voided urines from asymptomatic adult females. Most of the detected species were anaerobes, which SUC cannot detect (43).

Finally, Nickel and colleagues used polymerase chain reaction (PCR)-electrospray ionization mass spectrometry to assess midstream voided urine, detecting genera not typically identified or reported by SUC, including *Bifidobacterium, Staphylococcus, Lactobacillus, Propionibacterium*, and *Corynebacterium* (44). Note that some of the genera detected in these studies contain species considered to be commensals [normal members of a healthy balanced (eubiotic) microbiome]. Others, however, include known (universally accepted) emerging uropathogens (newly recognized and understudied), and/or suspected uropathogens.

## Evidence that detected bacteria are alive

**Figure 1:**
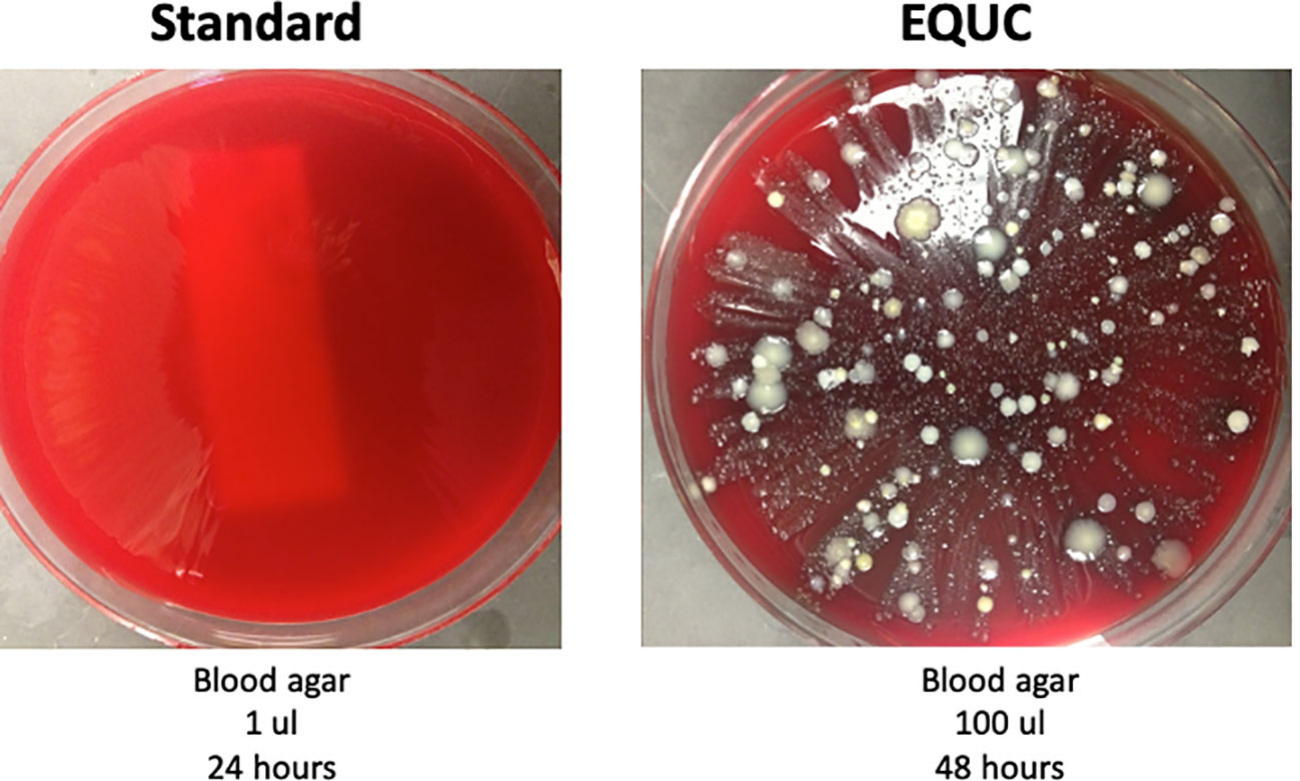
This urine is not sterile. Urine was obtained by transurethral catheter from a woman seeking urogynecology care. (Left) 1 μL was spread on a blood agar plate and incubated for 24 h at 35°C at ambient atmosphere. This is part of the standard urine culture (SUC) protocol. (Right) 100 μL was spread on a blood agar plate and incubated at 35°C for 48 h. This is one of many growth conditions that comprise the enhanced quantitative urine culture (EQUC) protocol. Reprinted with permission from College of American Pathologists. *CAP Today*, Aug. 2016.

Although these studies documented evidence of microbial DNA presence in urine, it was unclear whether the microbes were alive. Thus, several research teams set about to address this issue. First, Khasriya and colleagues conducted a large prospective study of males and females with and without chronic lower urinary tract symptoms, comparing SUC results with those of “sediment cultures,” performed on shed urothelial cells concentrated by centrifugation. The latter method detected many species missed by SUC, because it included growth conditions not used by SUC and because some of those species associate closely with shed urothelial cells. The authors recommended that the diagnostic algorithms and treatment plans for individuals with chronic urinary tract symptoms be re-evaluated (45). Using a different research strategy, Hilt and co-workers established an enhanced culture method called expanded quantitative urine culture (EQUC), plating 100× more urine than SUC (which uses 1 μL) on several different growth media under multiple atmospheric conditions and incubating for twice as long (48 h). These authors obtained urine by transurethral catheter from adult females with and without overactive bladders and compared the results obtained by SUC and EQUC. The vast majority of samples were deemed “no growth” by SUC using a threshold of 10^3^ CFU/mL (**Figure 1**). In contrast, EQUC detected bacteria in 80% of the samples. EQUC detected 35 different genera and 85 different species, most not identified by SUC (25). The same research team used an expanded version of EQUC to determine its most efficient or streamlined version. In the process, they calculated that SUC has a 90% false-negative rate for all bacterial species detected by EQUC, and a remarkably high 50% false-negative rate for all taxa currently accepted as uropathogenic (22). More recently, Hochstedler and colleagues reported a cross-sectional study that compared the SUC and EQUC results of paired urine samples obtained by transurethral catheterization and midstream voided urine from adult females with recurrent UTIs (46). EQUC detected considerably more unique bacterial species than SUC, including universally accepted uropathogens, from both catheterized and voided urine. Whereas SUC and EQUC equally detected *Pseudomonas* species and genera in the family Enterobacteriaceae (especially the accepted uropathogens *Escherichia, Proteus*, and *Klebsiella*), EQUC detected several emerging uropathogens that SUC did not. For example, SUC did not detect the emerging uropathogens *Actinotignum* (formerly *Actinobaculum*) *schaalii, Alloscardovia omnicolens*, or *Streptococcus anginosus*, and it drastically underreported the emerging uropathogen *Aerococcus urinae*. Moreover, it performed quite poorly on the accepted uropathogen *Enterococcus faecalis*. Furthermore, it failed to detect several established uropathogens, including *Staphylococcus aureus*, *Streptococcus agalactiae* (Group B Strep), and the yeast *Candida*. Other early publications also highlighted the limitations of SUC (47, 48), as have several recent ones. In addition to 16S rRNA gene sequencing (mentioned above), Vaughan and colleagues assessed their catheterized urine samples with EQUC, which detected microbes in approximately 60% of SUC-negative urine samples. Many of those microbes are accepted uropathogens (e.g., *E. coli, Pseudomonas. aeruginosa*, and *E. faecalis*), whereas others are emerging uropathogens (e.g., *A. schaalii, Aerococcus urinae*, and *S. anginosus*) and uropathogenic fungi (*Candida* and *Aspergillus*) (42). Likewise, in addition to sequencing, Perovic and colleagues used an enhanced culture approach similar to EQUC to assess their midstream voided urine samples. In total, they identified 297 bacterial species, including both accepted uropathogens (e.g., *E. coli, E. faecalis*, and *S. aureus*) and emerging uropathogens (e.g., *S. anginosus, A. schaalii*, and *A. urinae*). The median was 53 species per sample, considerably more than is often detected by SUC, which typically dismisses results with three or more microbes as contamination (43). Our recent calculation of results from 1,000 catheterized urine samples shows that EQUC detected more than 70% of all bacterial genera detected by 16S rRNA gene sequencing (49). The missing genera are mostly strict anaerobes that die upon exposure to oxygen (i.e., *Sneathia*) and bacteria with no cell wall (e.g., *Ureaplasma*). EQUC also does not grow obligate intracellular bacteria (e.g., *Chlamydia trachomatis* or *Mycobacterium tuberculosis*). Thus, the bacteria detected by DNA-based detection methods (e.g., 16S rRNA gene sequencing, shotgun metagenomic sequencing, and multiplex PCR) are alive and culturable, but most are not or are poorly detected by SUC.

## Evidence for association with lower urinary tract symptoms

The evidence accumulated to this point documented that DNA present in human urine was often related to living microbes. However, the mere presence of microbes did not shed light on the relationship to urinary symptoms. Thus, given that most bacteria identified by DNA sequencing could be cultured, the next issue was whether the species missed by SUC could cause symptoms.

One complication arose, as many species found in symptomatic participants were also found in asymptomatic controls. For example, *E. coli* was detected in adult females with UTI-like symptoms and in age-matched controls in a similar abundance, albeit less frequently (50, 51). However, early studies did note a correlation between lower urinary tract symptoms and urobiome composition. For example, Pearce and colleagues obtained catheterized urine from women with and without urgency urinary incontinence (UUI). Using both 16S rRNA gene sequencing and EQUC, they assessed these samples, detecting almost three times as many species in the UUI cohort than in the non-UUI controls. Whereas the genera *Lactobacillus* and *Streptococcus* were enriched in the non-UUI controls, several diverse taxa were enriched in the UUI cohort, including *Actinobaculum*, *Actinomyces, Aerococcus, Corynebacterium, Gardnerella, Staphylococcus*, and *Streptococcus.* At the species level, the UUI cohort was enriched for *Actinotignum* (formerly *Actinobaculum*) *schaalii, Winkia* (formerly *Actinomyces*) *neuii, Aerococcus urinae, Pseudoglutamicibacter* (formerly *Arthrobacter*) *cumminsii, Corynebacterium coyleae, Gardnerella vaginalis, Lactobacillus gasseri*, and *S. anginosus.* In contrast, only *L. crispatus crispatus* was enriched in controls (47). Because several studies that used EQUC to identify bacteria in catheterized urine were designed identically (22, 47, 51–56), Joyce and colleagues were able to re-analyze data from 1,004 adult females comprising 346 unaffected controls and 658 diagnosed with the following lower urinary tract symptoms: UTI (n=304), UUI (n=255), stress urinary incontinence (SUI) (n=50), and interstitial cystitis/painful bladder syndrome (n=49). After adjusting for age, the microbes detected in the UTI, UUI, and SUI cohorts differed significantly from those detected in the controls. In the UTI cohort, as expected, *Escherichia* was both prevalent and abundant but other microbes were also prevalent, albeit at more modest abundances. These included members of the genera *Lactobacillus, Streptococcus, Staphylococcus, Corynebacterium, Actinomyces*, and *Aerococcus*, raising the likelihood that members of these genera could contribute to UTI-like symptoms. These genera were also both prevalent and highly abundant in the UUI cohort, especially the emerging uropathogen *S. anginosus*. Intriguingly, the same taxa were also detected in controls, but at vastly lower levels of both prevalence and abundance. These results suggest that an overabundance of commensal bacteria could contribute to UUI-associated symptoms (57). Another recent study assessed catheterized urine obtained from adult females with acute uncomplicated UTIs or recurrent UTIs, comparing 16S rRNA gene sequencing with SUC. The former detected bacteria in significantly more samples than the latter (69.0% and 16.7%, respectively). Importantly, the bacteria detected in the two cohorts differed. Whereas the uncomplicated UTI microbiomes tended to be predominated by members of the family Enterobacteriaceae, especially *E. coli*, the recurrent UTI microbiomes were considerably more diverse, containing many genera detected in other studies including both accepted and emerging uropathogens (58). Note that while some of the UTI- and UUI-associated genera contain accepted uropathogens (e.g., *E. coli* and *S. aureus*), others contain emerging uropathogens and most of those are either not detected or are underreported by SUC (e.g., *A. urinae, A. schaalii*, and *S. anginosus*).

## Standard urine culture versus expanded quantitative urine culture

A recent report directly compared SUC and EQUC. Barnes and colleagues performed a clinical trial in which adult females who thought they had a UTI were randomized to either SUC or EQUC for diagnosis and treatment. The primary outcome, UTI symptom resolution determined 7–10 days following enrollment, did not differ significantly. However, the secondary outcome, symptom score assessed by the UTI Symptom Assessment (UTISA) questionnaire, differed significantly; patients diagnosed using EQUC experienced a greater decrease in the median symptom score. Importantly, in an exploratory analysis, the authors separated the patients predominated by *E. coli* from those predominated by non-*E. coli* species and compared symptom resolution. Although statistical significance was not reached, the trend toward greater symptom resolution in those diagnosed by EQUC suggests that further research with a larger sample size is warranted (56).

This trend was likely due to the documented ability of EQUC to detect uropathogens that SUC cannot. For example, Hochstedler and co-workers characterized the urobiome of adult females with recurrent UTIs, comparing urine collection and culture methods. They obtained paired catheterized and midstream voided urine samples and assessed them by both SUC and EQUC. Detection of microbes differed by both collection and culture method, with EQUC detecting more microbes than SUC in both urine sample types. EQUC detected UTI-associated microbes and unknown UTI-associated microbes [those not known to be beneficial (thus excluding *Lactobacillus*) and not known to be associated with UTI]. The proportion of cultures positive for UTI-associated microbes was considerably larger when assessed by EQUC than by SUC (catheterized: EQUC=53.5% and SUC=39.5%; voided: EQUC=90.5% and SUC=57.1%). This was also true for unknown UTI-associated microbes (catheterized: EQUC=11.6% and SUC=0%; voided: EQUC=66.7% and SUC=26.2%) and for non-UTI-associated microbes (i.e., *Lactobacillus*) (catheterized: EQUC=16.3% and SUC=2.3%; voided: EQUC=47.6% and SUC=2.4%). As such, the proportion of cultures without growth was smaller with EQUC (catheterized: EQUC=39.5% and SUC=65.1%; voided: EQUC=4.8% and SUC=28.6%). As noted in other studies, SUC often missed accepted (especially *E. faecalis*) and emerging uropathogens (including *A. schaalii*, *A. urinae*, and *S. anginosus*). Using the results from EQUC assessment of catheterized urine samples, the authors calculated sensitivity, specificity, and both positive and negative predictive values for species that are typically thought to be associated with UTI symptoms. For SUC assessment of voided urine, the presumed “gold standard”, the values for these variables were rather poor, ranging from 0.65 to 0.68. SUC assessment of catheterized urine was excellent for specificity (1.0) and positive predictive value (1.0), but poor for sensitivity (0.57) and negative predictive value (0.66). EQUC assessment of voided urine was excellent for sensitivity (0.91) and specificity (1.0), but poor for both predictive values (0.50 and 0.53). The authors concluded that EQUC is superior to SUC, and catheterized urine superior to voided urine, at least for this population (46). While these results cannot be directly generalized to a broader population, it is logical to assume that assessment by any assay that fails to detect many accepted and emerging uropathogens cannot be considered the “gold standard” or used to assess the value of another assay, whether culture or DNA based.

## The role of the standard urine culture

SUC is deeply ingrained in clinical medicine. Despite its limitations and those of screening microscopy, these tests will continue to be performed with clinical interpretations that are often flawed. Given that rapid replacement of these tests is unlikely, a realistic expectation is that clinicians raise their awareness of the well-documented limitations, especially in subgroups of patients where there is an altered risk-benefit relationship of antibiotic use. For example, patients with recurrent UTIs, and the associated repetitive antibiotic exposure, might benefit from a more comprehensive assessment of urobiome community members so that the clinician can be aware of the potential loss of beneficial microbes as a contributor to the underlying cycle of recurrent UTI. Going beyond a simple “kill the uropathogen” approach to restore a healthy or eubiotic urobiome may be as important, or perhaps even more important, than a course of antibiotic therapy. Although research is needed to define the various forms of eubiotic or healthy urobiomes, as well as the clinical techniques for eubiotic urobiome restoration, these ideas are certainly well within the capability of modern science. Moreover, the situation goes beyond understanding microbial ecosystem of the urinary tract. Until we fully understand host susceptibilities, and the intricate immune responses elicited by “shifts” in the urobiome, it may be difficult to pinpoint causation.

Better techniques would be especially important in medically vulnerable patients, such as those with recurrent, life-threatening *C. difficile* infections. As an example of microbiome restoration, *C. difficile* treatment with fecal microbial transplantation demonstrates the feasibility and supports the logic of microbiome restoration. It is in this vulnerable population where prudent use of systemic antibiotics is especially important. For individuals with recurrent UTIs who are prone to *C. difficile*, unquestioning reliance on an SUC seems ill advised. Instead, clinicians may wish to augment their testing with enhanced urine culture (which any hospital microbiology laboratory can perform) or either DNA sequencing or multiplex PCR (both available privately and within select health systems). Note, however, that sequencing will detect microbes about which we know almost nothing. Caution should be taken before embarking on a therapeutic strategy. The goal should not be to sterilize the urinary tract.

Finally, there is the group of suffering patients whose diagnosis remains elusive. Although their symptoms mimic a UTI, no microbial association or causation has been found at either the individual microbe or the microbial community level. The desperation of these patients and their clinicians often results in the initiation of treatments that do not have rigorous efficacy evidence. While it is unclear whether additional techniques for urine testing will be beneficial for these patients, reliance on SUC is rarely beneficial for their care. The role of antibiotic therapy in the presence of ongoing abnormally high urine leukocytes is an area of active investigation (59).

## Conclusion and summary of our thoughts

If one practices medicine long enough, one experiences how dogma changes as high-quality evidence becomes available and patient care improves. No longer do clinicians treat ulcers with a “bland” diet—instead, *H. pylori* treatment is initiated. No longer is cancer a single disease—instead, there is a growing appreciation of the genetic differences that allow oncologists to tailor treatment that is not solely based on an organ of origin. There are many examples of science informing clinical medicine.

We believe that sufficient urobiome science exists such that all clinicians can be aware of the limitations of SUC. The dogma of “no growth equals negative SUC” is incorrect. As the global crisis of antibiotic resistance grows, clinicians can learn new diagnostic and treatment algorithms that will refine the role of UTI antibiotic use. The growing urobiome research community has the opportunity to play a key role in generating the rigorous evidence needed to understand recovery from UTIs or other lower urinary tract disorders and techniques for restoration of a healthy eubiotic urobiome. While we await additional high-quality evidence, clinicians should step away from the simplistic interpretation of SUC. Society guidelines should update “UTI” diagnostic criteria and step away from the simplistic dichotomy that has governed this area of clinical medicine for decades. Instead, guidelines should capture the “uncertainty” that exists, and incorporate clinical contexts and available evidence. Finally, a definitive test is needed that is timely (i.e., takes hours) and accurately determines the microbiota present, assessing both relevant commensals and potential uropathogens. It must also respect antibiotic stewardship, suggesting treatment alternatives that treat the patient’s symptoms effectively.

## Author contributions

AJW conceived the manuscript. AJW and LB drafted the manuscript. All authors contributed to the article and approved the submitted version.
